# Novel Electromagnetic Heat Hydrodistillation for Extraction of Essential Oil from Tangerine Peel

**DOI:** 10.3390/foods13050677

**Published:** 2024-02-23

**Authors:** Na Yang, Yamei Jin

**Affiliations:** 1 State Key Laboratory of Food Science and Resources, Jiangnan University, 1800 Lihu Road, Wuxi 214122, China; yangna@jiangnan.edu.cn; 2 School of Food Science and Technology, Jiangnan University, 1800 Lihu Road, Wuxi 214122, China; 3 Collaborative Innovation Center of Food Safety and Quality Control in Jiangsu Province, Jiangnan University, 1800 Lihu Road, Wuxi 214122, China

**Keywords:** electromagnetic heat, essential oil extraction, oscillating magnetic field, physicochemical properties

## Abstract

A novel electromagnetic heat method is presented for green extraction of natural compounds from peel residue. In the processing cavity obtained through 3D printing, a core made of amorphous alloy was applied to strengthen the magnetic flux. During the process, an induced electric field was produced in the extract medium owing to an oscillating magnetic field at 50 kHz rather than a pair of electrodes; thus, electrochemical reactions could be avoided. A thermal effect and temperature rise were observed under the field, and essential oil was obtained via this electromagnetic heat hydrodistillation. In addition, the numerical relationships between magnetic field, induced electric field (IEF), induced current density, and temperature profile were elaborated; they were positively correlated with the extraction yield of essential oils. It was found that the waveforms of the magnetic field, induced electric field, and excitation voltage were not consistent. Using a higher magnetic field resulted in high current densities and terminal temperatures in the extracts, as well as higher essential oil yields. When the magnetic field strength was 1.39 T and the extraction time was 60 min, the maximum yield of essential oil reached 1.88%. Meanwhile, conventional hydrodistillation and ohmic heating hydrodistillation were conducted for the comparison; all treatments had no significant impact on the densities. In addition, the essential oil extracted by electromagnetic heat had the lowest acid value and highest saponification value. The proportion of monoterpenoids and oxygen-containing compounds of essential oil extracted by this proposed method was higher than the other two methods. In the end, the development of this electromagnetic heat originating from magnetic energy has the potential to recover high-value compounds from biomass waste.

## 1. Introduction

Citrus is the world’s most abundant fruit; it is widely grown in tropical and subtropical regions, constituting 60% of global fruit yield [1]. Depending on the variety of citrus, the peel comprises 30 to 60% of the fruit’s weight. In addition, citrus peel is composed of irregular parenchyma cells, enclosing numerous glands or oil sacs [2,3], and is the primary source of essential oil. The volatile components in plant oils constitute predominantly terpenes and their derivatives (limonene, linalool, and citronellol), which make up 85 to 99% of the active ingredients [4,5]. Owing to its inherent antioxidative, anticancer, and broad-spectrum antibacterial properties, essential oil from citrus is often used as a natural preservative, nutritional supplement, and flavoring compound in cosmetic, pharmaceutical, and food industries [6]. The global essential oil market continues to grow, which is projected to exceed 3200 million USD by 2025, with citrus essential oil claiming to be the largest market share [7]. The overall yield of *Nanfeng* tangerine (*Citrus reticulata* Blanco cv. Kinokuni) has increased in recent years because of the continuous expansion of the cultivation area as well as the advancement of processing technology, resulting in the generation of massive amounts of tangerine peels each year, which cause serious environmental pollution [8]. Therefore, it is of great importance to utilize these byproducts to transform them into value-added products for the sustainability of citrus industries and environmental protection.

The essential oil content in tangerine peels reaches up to 4%. The extraction process is the critical factor in determining the yield and quality of citrus essential oil. However, conventional extraction methods, such as steam distillation and hydrodistillation [9], use heat or pressure to increase internal pressure in essential oil-bearing plant cells to destroy the walls and release essential oil [10], which leads to a long extraction time, high energy consumption, as well as loss of heat-sensitive and volatile components. For instance, the yield of citrus essential oil from sour orange peel was only 0.25% using conventional hydrodistillation for 6 h [11], indicating a low extraction efficiency. In addition, cold pressing is a traditional extraction method. The oil sacs or glands of citrus peels break and release essential oils in a water emulsion during mechanical cold pressing and are centrifuged to recover the essential oils [12]. However, essential oils prepared by cold pressing can contain some non-volatile components, such as coumarins, psoralens, and polymethoxyflavonoids, a class of oxygen heterocyclic compounds, which are phototoxic and may pose a risk to the body when ingested in large quantities [13].

In recent years, non-conventional extraction technologies, such as ohmic heating extraction and microwave heating extraction, have begun to emerge. Microwave heating extraction uses microwave radiation to heat the extract and solvent mixture; such a phenomenon facilitates the speeding up of the extraction for active components, improving the yield of essential oil. For example, microwave-assisted extraction of essential oil from citrus peel achieved a yield of 10.47 to 11.5% compared to the 5.95% yield for hydrodistillation under the same conditions [14]. However, microwave heating sometimes results in changes in the chemical structure of some components of essential oil [15]. Ohmic heating extraction treats the extract as a resistor. When the current released by the electrode passes through the medium, electrical energy is converted into the heat within it, causing a rapid temperature rise. Concurrently, the electric field causes the electroporation of cell membranes as well as the destruction of cell walls, increasing membrane permeability and improving extraction efficiency. For example, the yield of 3.6% was reached by using ohmic heating for 58.4 min during the extraction of essential oil from lemon peels [16]. However, citrus peel-like biomass is heterogeneous in nature, with varying impedances across different areas, which leads to uneven volumetric heating. Furthermore, electrode corrosion in multiphase electrolyte solution is a significant obstacle to the industrial application of ohmic heating extraction [17].

Therefore, we previously used magnetic energy to induce an electric field inside a conductive liquid and constructed a novel electromagnetic heat apparatus [18]. It is an emerging extraction technology based on the transformer principle. During the process, the induced electric field (IEF) originated from an oscillating magnetic field, and it does not require the use of electrodes. Moreover, it produces uniform heat distribution throughout the medium under the action of induced current. This method was successfully applied to the extraction of essential oils from citrus peel [19] and yellow horn seeds [20], which showed an improvement in the yield of essential oils and a reduction in extraction time.

However, the detailed principles with processing parameters have not been illuminated yet, especially in the relationship between applied magnetic field and induced electric field, as well as its waveform. Therefore, the aim of this study is to investigate this electromagnetic heat hydrodistillation (EH-HD) and its characteristics for essential oils extraction from *Nanfeng* tangerine peel. The influence of magnetic field strength on induced current, extraction yield, terminal temperature, as well as energy efficiency were evaluated. By comparing conventional hydrodistillation (HD) and ohmic heating hydrodistillation (OH-HD), the essential oil’s physicochemical properties containing the density, moisture content, saponification, acid value, volatile components, and microscopic morphology of the residue were investigated because the other extraction apparatuses are consistent with EH-HD in terms of condensation reflux and essential oil collection. The difference between HD, OH-HD, and EH-HD lies in the heating source. HD heating relies on thermal conduction, OH-HD relies on the electric field between the electrodes, while EH-HD lies in the oscillating magnetic field.

## 2. Materials and Methods

### 2.1. Materials and Reagents

Tangerine peels provided by Huiyuan Juice Group (Chengdu, China) were used as the raw material. Firstly, the peels were cleaned to remove impurities. They were naturally dried for 2 days at an ambient temperature of 30–35 °C and 30% relative humidity. They were then crushed and ground, followed by sieving through a 0.25 mm mesh. The powder was further dried at 85 °C until constant weight and stored in a desiccator for subsequent use. All the chemicals used were of analytical grade.

### 2.2. Apparatus Description

**Figure 1:**
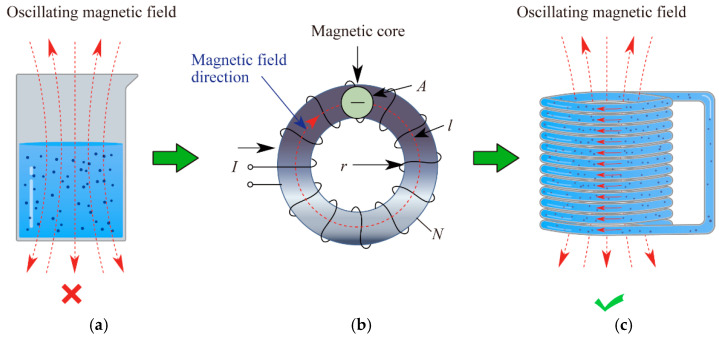
Principle of novel electromagnetic heat hydrodistillation on extract solution. (**a**) Extracts in a container subjected to the magnetic field; (**b**) winding coil on a magnetic core excited by the current; (**c**) extracts in a spiral winding subjected to the magnetic field.

Most extract solutions have specific conductivity and belong to a complex electrolyte system. However, there is no thermal effect on the solution since it is subjected to an oscillating magnetic field directly. Because the induced voltage could not drive the directional migration of charged solutes within the medium, the induced current to heat it is not established (Figure 1a). However, the electromagnetic effect still exists on the solution owing to the oscillating magnetic field (Figure 1b,c). Through preliminary experiments, a magnetic core was used to enhance the applied magnetic field, and then the thermal effect was observed. It indicated that magnetic field strength, sample winding, and sample conductivity are key factors for the heating [21].

**Figure 2:**
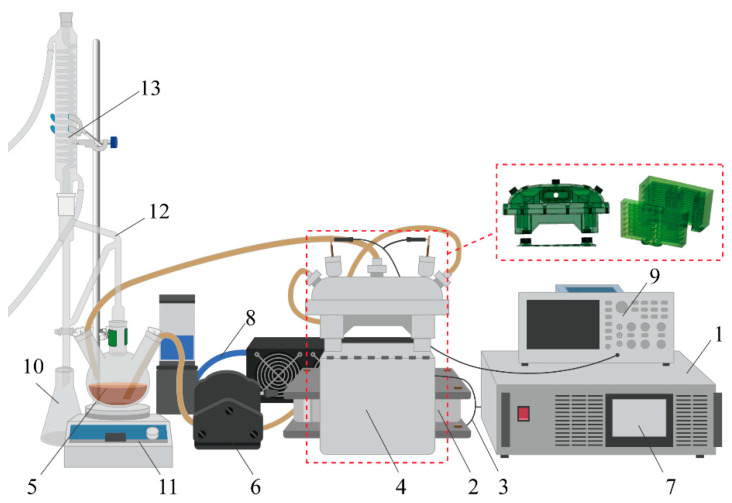
Electromagnetic heat hydrodistillation apparatus for the essential oil extraction. 1. Power source, 2. Magnetic core, 3. Primary winding, 4. Secondary winding of pipeline (3D printing material: PC-ISO, Stratasys, Eden Prairie, MN, USA), 5. Sample bottle, 6. Pump, 7. Control panel, 8. Cooling system, 9. Oscilloscope, 10. Collecting bottle, 11. Magnetic stirrer, 12. Essential oil receiver, 13. Condenser tube.

Thus, the apparatus consisted of a power source (INDUC Scientific Co., Ltd., Wuxi, China), magnetic core, primary winding (*N*_1_ = 1, litz wire), secondary winding (*N*_2_ = 24, PC-ISO supporting spiral tube of sample pipeline, length: *L* = 750 cm, inner diameter: *d* = 8 mm, and wall thickness: *d_w_* = 1.5 mm), sample bottle, pump, cooling system, and oscilloscope. A schematic diagram of the apparatus is presented in Figure 2. The power source provided rectangular voltage with 50–1000 V_amp_ (*U*_1_) at 50 kHz with 50% duty cycle (*D*). The secondary winding was manufactured via the industrial 3D printer (Fortus 450 mc, Stratasys, Edina, MN, USA). The magnetic core was made of amorphous Fe-Si-Ge alloy; its effective circuit length and cross-sectional area were 0.68 m and 36 cm^2^, respectively. The saturation magnetic flux density (*B_S_*) of the core was 1.50 T, and the excitation voltage *U*_1_ primary current (or excitation current) with or without the load, namely *I*_1_ and *I*_0_, was recorded via the power source.

Based on Ampere’s loop law and transformer principle [22], the relationship between excitation voltage (*U*_1_) and produced magnetic flux density (*B*), as well as induced voltage (*U*_2_), IEF strength (*E*), induced current (*I*_2_), and induced current density (*J*) loaded on the sample are shown in Equations (1)–(5):

(1) $$U_{1}=4fSN_{1}B$$

(2) $$\frac{U_{1}}{U_{2}}=\frac{N_{1}}{N_{2}}$$

(3) $$E=\frac{U_{2}}{L}$$

(4) $$I_{2}=\frac{U_{1}\cdot (I_{1}-I_{0})}{U_{2}}$$

(5) $$J=\frac{I_{2}}{S_{P}}=\frac{4I_{2}^{2}}{\pi d^{2}}$$

where *L* is the length of the winding coil. *S* and *S_P_* are the cross-section areas of the magnetic core and winding coil, respectively. *I*_0_ is the unloaded current at a primary voltage. *N*_1_ and *N*_2_ are the number of turns of the primary and secondary winding.

The energy efficiency of the extraction (*η*) was evaluated based on the proportion of input power (*P*_1_) to output power of the winding of the medium (*P*_2_). These equations are described as Equations (6)–(8):

(6) $$\eta =\frac{P_{2}}{P_{1}}=\frac{I_{2}U_{2}}{I_{1}U_{1}}$$

(7) $$I_{1}=\Delta I+I_{0}$$

(8) $$P_{2}=I_{2}\cdot U_{2}=\Delta I\cdot U_{1}$$

### 2.3. Electromagnetic Heat Hydrodistillation (EH-HD), Hydrodistillation (HD), and Ohmic Heating Hydrodistillation (OH-HD)

The liquid sample (20 ± 1 °C) was prepared with 100 g peel powder dissolved in deionized water. The solid–liquid ratios of the extraction were 1:10 (*w*/*v*), 1:20 (*w*/*v*), 1:30 (*w*/*v*), and 1:40 (*w*/*v*). The conductivity of the sample solution was adjusted by 0.1 M NaCl solution at 10 S/m, according to the literature, with slight modification [23]. Then, it was pumped into the sample winding at a flow rate of 10 L/h for a cyclic flow, and the duration was 60 min. The power source was operated at the excitation voltages *U*_1_: 50–1000 V_amp_. In this case, the induced current was loaded on the winding of the fluid for the heating. Then, the temperature profile of the extract solution was measured via an infrared thermometer (H10, Hikmicro Co., Ltd., Hangzhou, China), which was located at a distance of 15 cm. The excitation voltage, as well as induced voltage (*U*_2_) loaded on the medium, were determined via a pair of platinum electrodes connected to the oscilloscope (TBS1102X, Tektronix Co., Ltd., Beaverton, OR, USA). The vapor entered the separator from the elbow tube, after which it was liquefied and condensed by the cooling system. After standing for one hour, the essential oil was collected from the separator, dried with anhydrous sodium sulfate, and stored at 4 °C for subsequent analysis.

The other extraction apparatuses are consistent with EH-HD in terms of condensation reflux and essential oil collection. Hydrodistillation was similar to EH-HD, except for electric stove heating (400 W) and time (3 h). In comparison, the duration and electric field strength used for ohmic heating hydrodistillation was 60 min and 25 V/cm, respectively. The extraction yield was calculated as follows:

(9) $$Y=\frac{v}{m}\times 100$$

where *Y* is the extraction yield (mL/100 g dw), and *v* and *m* are the volume of extracted essential oil (mL) and masses of raw materials (g), respectively.

### 2.4. Essential Oil Characterization

#### 2.4.1. Essential Oil Density

A volumetric flask (10 mL) was dried in an oven at 105 °C until constant weight. The weight of the dried volumetric flask (*W_I_*) was recorded accurately. Then, the flask was filled with essential oil to the scale line, and its weight (*W_F_*) was recorded again. The density of the oil was calculated as follows:

(10) $$Density\;(g/mL)=(W_{F}-W_{I})/10$$

#### 2.4.2. Moisture Content

An evaporating dish was dried in an oven at 105 °C until constant weight. Using an analytical balance, 2.000 g of essential oil was weighed, and the dish was placed in a glass desiccator to dry the moisture from the oil. The weight was taken every half an hour until it stopped fluctuating. The moisture content was determined according to the weight difference between the extracted essential oil and dried essential oil.

#### 2.4.3. Acid Value

A weight (*m* = 2.5 g) of essential oil was taken in a 100 mL beaker. In another beaker, 1% phenolphthalein was prepared using anhydrous ethanol. In the sample beaker, 0.5 mL phenolphthalein indicator was added, and the mixture was titrated with potassium hydroxide (KOH) at a concentration of 0.1 mol/L (*C*) when the beaker was over an electric stove to heat the mixture. The volume of potassium hydroxide (*V*) required to turn the solution pink–red was recorded. The acid value (*AV*) was calculated using the following formula:

(11) $$AV=\frac{56.1\times C\times V}{m}$$

where 56.1 is the molecular weight of KOH.

#### 2.4.4. Saponification Value

The saponification value (*SV*) is the quantity of potassium hydroxide (KOH) required to saponify 1 g of fat completely. About 2 g (*m*) of essential oil was mixed with 25 mL of 1 mol/L potassium hydroxide–ethanol solution (KOH–EOH) in a 250 mL flask. Then, the mixture was refluxed with a condenser attached to a water bath for 1 h. The resulting solution was cooled and titrated with 0.5 mol/L (*C*) hydrochloric acid (HCl) using 0.5 mL of phenolphthalein indicator until colorless, and the volume of HCl was recorded as *V*1. A blank test was conducted by replacing the essential oil with distilled water; under the same conditions, the quantity of consumed HCl was recorded as *V*0. The saponification value was calculated as follows:

(12) $$SV=\frac{(V0-V1)\times C\times 56.1}{m}$$

where 56.1 is the molecular weight of KOH.

#### 2.4.5. GC-MS Analysis

The component of extracted essential oils was determined according to the procedure developed by Hashemi et al. [24] with slight modification. In brief, 0.1 g essential oil was diluted with petroleum ether 10 mg/mL, which was filtered through 0.22 μm as the sample for GC-MS analysis via a chromatographic column of TG-5MS (30 m × 0.25 mm × 0.25 µm film thickness). The gap chromatographic conditions were as follows: the initial temperature was 50 °C, which increased at the interval of 8 °C/min to 160 °C; held at 160 °C for 8 min; then increased from 160 °C to 260 °C at the interval of 8 °C/min; and held at 260 °C for 12 min. The carrier gas was helium with a flow rate of 1 mL/min; the injector temperature was 220 °C; the sample injection volume was 1 µL; the split ratio was 20:1. Mass spectrometry conditions: electron ionization (EI) mode with an ionization voltage of 70 eV; ion source temperature and transmission line temperature of 280 °C; solvent delay of 2.5 min; scanning range of 20–600 amu.

After GC-MS analysis, the ion chromatogram (TIC) of essential oil was obtained. The components were analyzed using the NIST98 mass spectrometry data retrieval library. The obtained spectra were manually verified and confirmed with a similarity greater than 80% as the basis for structural confirmation. The relative mass fractions of each component were calculated using the area normalization method.

### 2.5. Scanning Electron Microscope (SEM) for Feedstock

The morphological analysis was conducted according to the method described by Haule et al. [25] with slight modification. Briefly, the dried granule was fixed on the sample holder using double-sided tape. After gold sputtering under vacuum, the morphology of the residue was observed using a scanning electron microscope SU8010 (Hitachi High-Technologies Co., Tokyo, Japan) at a magnification of 5000× with an accelerating voltage of 5 kV.

### 2.6. Statistical Analysis

All experiments and measurements were repeated at least three times. The IBM SPSS Statistics v.25.0 (SPSS Inc., IBM Co., Armonk, NY, USA) was used for statistical analysis, and the Origin 2019 software (Origin Lab., Northampton, MA, USA) was utilized for graphic drawing. All data were presented as mean value ± standard deviation (SD).

## 3. Results and Discussion

### 3.1. Process Parameters

**Figure 3:**
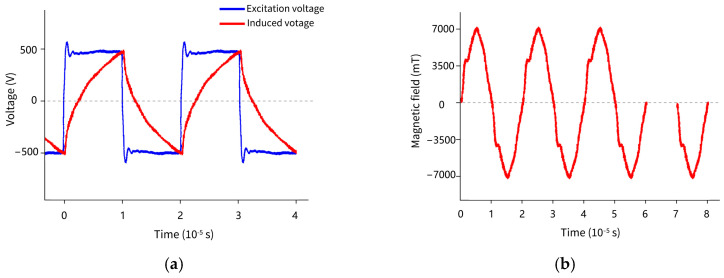
Waveforms sampling. (**a**) Excitation voltage and induced voltage (or IEF loaded on extracts solution); (**b**) the produced oscillating magnetic field.

In this study, the electric field in the sample solution was induced by an oscillating magnetic field during the electromagnetic heating process. As shown in Table 1, the produced magnetic field and electric field loaded on the extracts increased as the excitation voltage improved. The IEF strength depended on the induced voltage that appeared in the sample winding. When the excitation voltage was 500 and 1000 V, the magnetic field strengths were 0.69 T and 1.39 T. Then, the theoretical and measured IEF values inside the sample were 16 V/cm and 13.5 V/cm, as well as 32 V/cm and 28.7 V/cm, respectively. Plant peels and stems contain components such as essential oils, pectin, minerals, cellulose, and water-soluble salts, which confer a certain electrical conductivity. Under the electric field, ion migration is promoted, leading to the generation of ion current and a rapid temperature increase in the solution. Coffee beans exhibit a temperature rise rate six times higher at 16 V/cm than that at 8 V/cm [26], similar to the results observed in thyme [27]. Palm fruit reaches 100 °C in just 20 s at 150 V; meanwhile, the heating rate accelerates with an increasing voltage gradient [28]. In this process, the induced current was loaded into the extract medium, and the thermal effects and energy consumption were thus observed. At excitation voltages of 500 and 1000 V, the induced currents were 0.14 A and 0.28 A. In practical equipment, the use of rectangular waves for the excitation has a high-cost performance. Therefore, a spike pulse wave of the produced magnetic field was observed simultaneously with the measurement of the induced voltage waveform on the extract solution, which was manifested as a sawtooth waveform (Figure 3). It also indicated that all three waveforms had differences, but the frequency was consistent at 50 kHz. A high change rate of magnetic flux was conducive to producing a higher electric field in the medium, which was presumed to have a better extraction effect.

**Figure 4:**
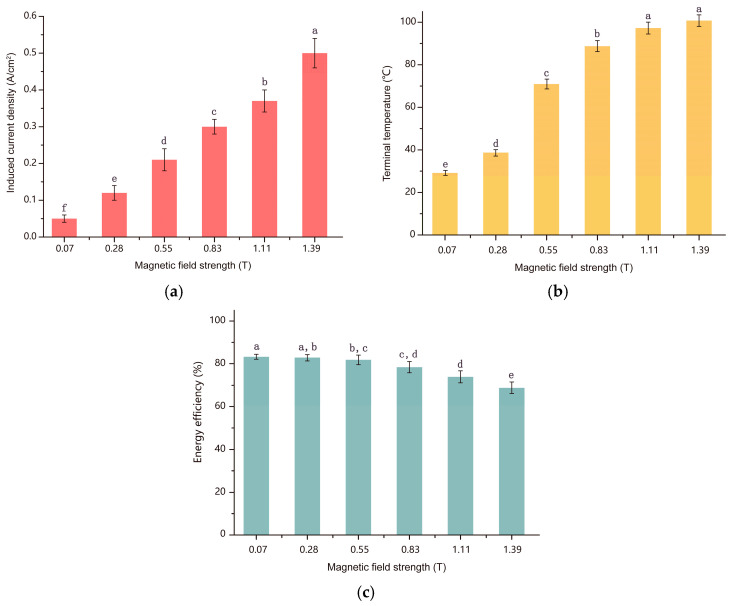
Influence of the magnetic field on the winding of extract solution at a solid–liquid ratio of 1:20. (**a**) Induced current density within the extract solution; (**b**) terminal temperature for retention time 60 min; (**c**) energy efficiency of the process. The different letters indicate significant differences at a 0.05 level.

Actually, the temperature is linearly related to the current density in the liquid [29]. During electromagnetic heat, as the applied magnetic field rose, the induced current density produced in the sample medium was increased based on Ampere’s loop law and Ohm’s law (Figure 4a). It had a positive correlation with the temperature rise during resistance heating. As shown in Figure 4b, the terminal temperature of the medium increased as the current density was improved. With an increase in voltage gradient, the resistance to current flow decreases, leading to heat generation and facilitating the rupture and softening of plant cell membranes. It promotes the extraction of essential oil components [30]. However, with the increase in an applied magnetic field, the heating efficiency was decreased (Figure 4c); it was because the magnetic flux in the core reached a saturation level, and the input of more energy would be dissipated in the form of extra heat, so it led to the decline in the efficiency of this extraction process. Therefore, in subsequent engineering designs, a larger core cross-sectional area is conducive to increasing the magnetic flux and energy power.

### 3.2. Effect of EH-HD Process Parameters on Extraction Yield

**Figure 5:**
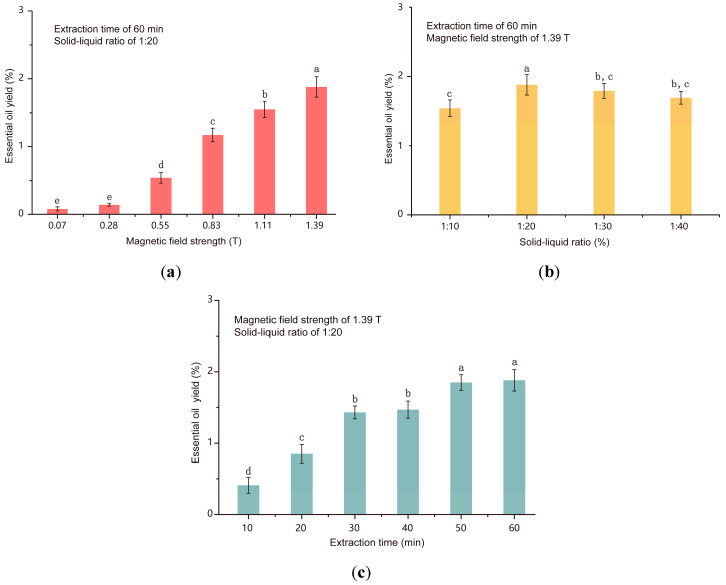
Influence of the proposed electromagnetic heat on essential oil yield from tangerine peel. (**a**) Magnetic field strength; (**b**) solid–liquid ratio; (**c**) extraction time. The different letters indicate significant differences at a 0.05 level.

Figure 5a–c illustrates the effects of the applied oscillating magnetic field, solid–liquid ratio, and extraction time on essential oil yield. It was observed that with an increase in magnetic field strength and an extension of the duration, the essential oil yield was increased. The maximum yield of 1.88% was achieved when the treatment time was 60 min, and the applied magnetic field was 1.39 T (Figure 5a). The principle behind this electromagnetic heating of pomace is based on the time-varying electric field induced within the medium. It facilitates mutual friction and collision between free ions and biomass, accelerating the overflow and dissolution of essential oil substances from plant cell walls into the reagent [19]. High magnetic fields lead to significant Joule heating within the extracts. Under the same pH conditions, different solid–liquid ratio mediums possess varying levels of electrical conductivity. Moreover, higher sample conductivity leads to faster temperature rise rates as well as higher terminal temperatures. During the extraction, as the extracts were released, the viscosity of the medium increased, thereby accelerating the heating. As shown in Figure 5b, it was suggested that different solid–liquid ratios of the sample solution exhibited differential solid content and viscosity; under the field, soluble solids in the medium resulted in higher essential oil yields. Since the electric field originated from oscillating magnetic fields, there was no need for electrodes, thereby avoiding adverse effects such as electrochemical corrosion and metal ion leaching during the heating process. In addition, the extraction time was directly proportional to the yield (Figure 5c).

Essential oils are complex and volatile compounds with functional activity. Common extraction methods include steam distillation; for example, grapefruit peel mixed with water at a 1:2 ratio and extracted at 140 °C for 180 min yields 1.4% essential oil with low limonene content [31]. Similarly, eucalyptus leaves distilled at a solid–liquid ratio of 1:5 yield only 1.19% essential oil [32]. High-temperature distillation can lead to the degradation of active components in essential oils, affecting both yield and quality. In recent years, electric fields, as an emerging technology, have been successfully applied to assist in the extraction of plant essential oils. For instance, honeysuckle mixed with a 1% NaCl solution at a solid–liquid ratio of 1:6 and extracted under 220 V for 26 min obtained essential oil with a yield similar to that of 1 h hydrodistillation, approximately 0.7% [33]. Similarly, under the same conditions, peppermint yields 2.29% essential oil [34]. However, sweet orange blossoms treated with a 1:4 NaCl solution under ohmic heating for 50 min yield only 0.047% essential oil [35].

### 3.3. Physicochemical Properties of Essential Oils

The physicochemical properties of essential oils extracted by conventional hydrodistillation (H-EO), ohmic heating hydrodistillation (OH-EO), and electromagnetic heat hydrodistillation (EH-EO) were analyzed. As shown in Table 2, the densities of essential oils after different extraction methods were 0.852 g/mL, 0.858 g/mL, and 0.849 g/mL, respectively, showing a slight difference in these levels, most probably because the differential composition of monoterpenoids and sesquiterpenoids, resulting in the loss of compounds with a lower boiling point after high-temperature extraction, which ultimately led to changes in essential oil densities. In the case of acid value, EH-EOs showed a lower level of 9.6% and 4.3%, respectively, when compared with H-EOs and OH-EOs. The lower the acid value, the better the purity and quality of the essential oil, while the high acid value indicates the presence of more acidic substances and the easier oxidative deterioration of the oil. In terms of saponification value, EH-EOs showed a higher level by 4.00% and 1.79%, respectively, when compared with that of H-EOs and OH-EOs. It might be that the obtained fatty acids in EH-EOs were smaller, enhancing the hydrophilic ability, while the impurity content was lower.

### 3.4. GC-MS Analysis

Citrus essential oil contains about 400 complex mixtures of compounds, consisting of 85–90% volatile components and 1–15% non-volatile components. The volatile components are mainly monoterpenoids and sesquiterpenoids, as well as their oxygen-containing derivatives [36]. Tangerine is a type of citrus; the components of the tangerine essential oil include 53 different compounds, among which 50, 52, and 49 compounds were detected in H-EOs, OH-EOs, and EH-EOs, respectively. There were significant differences in the relative contents of each component of tangerine essential oil extracted by the three methods, which was consistent with the literature by Değirmenci and Erkurt [37]. Compared with the other two essential oils, EH-EOs showed less diverse compounds; however, the proportion of active components, such as *D*-limonene and limonene, was up to 63.54%, which was 17.28% and 6.43% higher than that of H-EOs and OH-EOs, respectively. Previous literature reported that *D*-limonene, limonene, γ-terpinenen, sabinene, α-pinene, β-pinene, β-myrcene, linalool, and α-terpinol were the main components of citrus peel essential oil [38].

In Table 3, the main ingredients in H-EOs were D-limonene (29.89%), limonene (24.29%), γ-terpinenen (13.61%), and 2-methylpentane (6.95%); those of OH-EOs were limonene (33.25%), D-limonene (26.45%), γ-terpinenen (9.29%), and sabinene (4.69%); while the main ingredients in EH-EOs were D-limonene (37.20%), limonene (26.34%), γ-terpinenen (10.11%), and sabinene (5.71%). During the extraction process, different proportions of chemical components may be produced, especially thermal and hydrolysis reactions, isomerization, polymerization, and thermal rearrangement [39]. D-limonene is an isomer of limonene; electromagnetic heat might accelerate the isomerization of eugenol to isoeugenol. Meryem et al. [40] showed that the main components of essential oils extracted by hydrodistillation from *C. limonum*, *C.paradisi*, and *C.reticulata* were, respectively, limonene (71.72%), α-pinene (6.23%), and geraniol (1.55%); limonene (56.31%), laurene (35.83%), and sabinene (2.78%); and limonene (76.5%), ρ-cymene (16.7%), and β-laurene (2.29%). Franco-Arnedo et al. [41] obtained antioxidant extracts from tangerine peels by supercritical CO_2_ extraction, which had high total phenol content, total flavone content, and in vitro antioxidant capacity. Eight kinds of flavonoids were tentatively identified: naringin, hesperidin, naringin, sinensetin, nobiletin, tetramethyl-o-scutellarin, heptamethoxyflavone, and tangeretin. The reasons for the difference in components were the differential extraction process and the kinds of citrus used as the raw material. For instance, the essential oils were rich in Limonene: 73.45% and 81.23% for conventional hydrodistillation (HD), as well as 75.91% and 86.14% for IEF-HD in grapefruit and pomelo peels, respectively [19]. Additionally, Tunç et al. [16] identified 25 and 24 chemical components from lemon waste essential oils by the conventional heating extraction/hydrodistillation (CHE/H) method and ohmic heating assisted extraction/hydrodistillation (OHAE/H) method, respectively. These differences were attributed to the difference in the extraction process.

The proportion of monoterpenoids in EH-EOs was the highest (95.37%) among the three essential oils. Compared with the H-EOs, the contents of oxygen-containing monoterpene and oxygen-containing sesquiterpene compounds in EH-EOs were significantly increased. The reason might be that an oscillating magnetic field accelerated the separation and extraction of oxygen-containing molecules, resulting in their higher contents. There was a slightly smaller difference between EH-EOs and OH-EOs in the proportion of oxygen-containing monoterpenes (7.86% and 7.18%, respectively). Miloudi et al. [42] showed that the electric field would accelerate the frequency of molecular collision, resulting in changes in the essential oil components. During the hydrodistillation, the electric field thus increases the transfer rate of solutes as well as accelerates the evaporation of volatile components from plant cells. In addition, electromagnetic waves are conducive to the separation of oxygen-containing components; for example, oxygen-containing components are separated under microwave action, which can increase the content of oxygen-containing compounds [43]. Oxygen-containing compounds make essential oils have unique fragrances and higher antioxidant activity [44], resulting in a potential application value that is higher than terpenoids. In this study, the electromagnetic heat resulted in a high content of oxygen-containing compounds (10.02%) among these extraction methods.

### 3.5. Morphological Analysis of Citrus Peel Residue (SEM)

**Figure 6:**
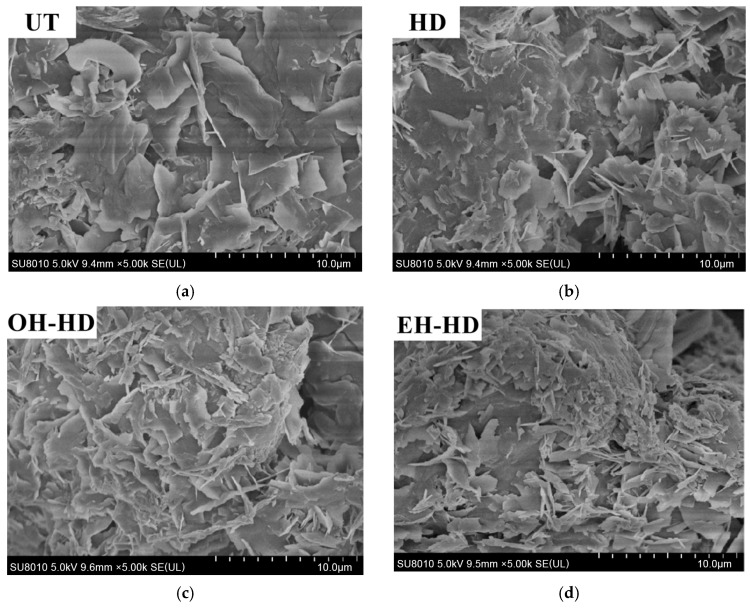
Scanning electron microscope (5000×) images of tangerine peel after different treatments. (**a**) Untreated; (**b**) conventional hydrodistillation; (**c**) ohmic heating hydrodistillation; (**d**) electromagnetic heat hydrodistillation.

Figure 6 shows the surface morphological changes of citrus peel residues before and after extraction by different methods. At a magnification of 5000×, the surface of untreated citrus peel residue was relatively regular and flat with a smooth tissue structure (Figure 6a). The extraction treatments had significant impacts on the surface morphology of citrus peel residues (Figure 6b–d). Both conventional heat treatment and electric field treatment led to cell wall rupture and separation; however, the surface fragmentation of citrus peel residues after ohmic heating hydrodistillation and electromagnetic heat hydrodistillation was more obvious, resulting in finer fragments. It showed that the non-thermal effect combined with the heating at the same temperature was conducive to destroying the cell wall and releasing more essential oil. This was similar to the literature of Chouhan et al. [45] about lemon peel tissues, where cell membrane structures were broken down, promoting the extraction of essential oils. Dereboylu et al. [46] observed that the electric fields enhanced tissue damage, thus making the extraction process more intensive. In previous studies, the essential oil was extracted by a magneto-induced electric field; it enhanced the permeability of the cell membrane due to the action of an oscillating magnetic field as an additional non-thermal effect, thus improving the diffusion of the essential oil and the extraction efficiency [20].

## 4. Conclusions

In this study, a novel electromagnetic heating method was demonstrated and applied to the extraction of essential oil from tangerine peel residue. The engineering characteristics and parametric formulas of the method were presented in detail for calculating excitation voltage, magnetic field strength, induced electric field, induced current, and heating efficiency. It was found that when rectangular wave excitation was applied, the measured magnetic field and induced electric field waveforms were all significantly different. During the extraction, the excitation source was an oscillating magnetic field, which generated an induced electric field for indirect heating, thus avoiding the issue of electrode corrosion and sample contamination. By comparing ohmic heating hydrodistillation using an electric field, the process was green and environmentally friendly. There was a positive correlation between magnetic field strength and the extraction rate of essential oils. After 60 min of electromagnetic heat treatment, the essential oil could reach its maximum extraction rate at an appropriate solid–liquid ratio. The surface morphology of the tangerine peel residue indicated that this novel electromagnetic heat process had caused serious damage to the raw material. Compared to other methods containing steam hydrodistillation and ohmic heating hydrodistillation, essential oils with higher yield and quality could be obtained in a shorter extraction duration. Then, it suggests the use of continuous processing and modular assembly of magnetic circuits is conducive to achieving high-throughput natural compound extraction.

## Figures and Tables

**Table 1:** Influence of excitation voltage on the magnetic field, induced voltage, IEF, and induced current *I*_2_ within the extract solution of tangerine peel.

Excitation Voltage (V)	Magnetic Field Strength (T)	Induced Voltage (V)	IEF-Calculated (V/cm)	IEF-Measured (V/cm)	*I*_2_ (A)
500	0.69	12,000	16	13.5 ± 0.2 ^b^	0.14
1000	1.39	24,000	32	28.7 ± 0.3 ^a^	0.28

Note: Lowercase letters with different superscripts in the same row indicate significant differences at a 0.05 level.

**Table 2:** Physicochemical properties of essential oils extraction from tangerine peel by different methods.

Physicochemical Properties	Conventional Hydrodistillation (HD-EO)	Ohmic Heating Hydrodistillation (OH-EO)	Electromagnetic Heat Hydrodistillation (EH-EO)
Densitiy (g/mL)	0.852 ± 0.003 ^b^	0.858 ± 0.001 ^a^	0.849 ± 0.002 ^b^
Acid value (mg KOH/g)	3.376 ± 0.022 ^a^	3.189 ± 0.018 ^b^	3.052 ± 0.017 ^c^
Saponification value (mg KOH/g)	189.31 ± 0.12 ^c^	193.42 ± 0.19 ^b^	196.88 ± 0.11 ^a^

Note: Lowercase letters with different superscripts in the same row indicate significant differences at a 0.05 level. HD conditions: temperature 100 °C, duration 3 h, solid–liquid ratio 1:20; OH conditions: electric field 25 V/cm, temperature 100 °C, duration 60 min, solid–liquid ratio 1:20; EH conditions: oscillating magnetic field 1.39 T, temperature 100 °C, duration 60 min, solid–liquid ratio 1:20.

**Table 3:** Ingredients of essential oils extracted from tangerine peel by different methods.

No.	Compound Name	Chemical Formula	Conventional Hydrodistillation (HD-EO)		Ohmic Heating Hydrodistillation (OH-EO)		Electromagnetic Heat Hydrodistillation (EH-EO)	
			Retention Time (s)	Area (%)	Retention Time (s)	Area (%)	Retention Time (s)	Area (%)
1	α-Pinene	C_10_H_16_	273.28	3.07	273.25	2.60	273.17	2.17
2	Camphene	C_10_H_16_	320.10	0.06	319.79	0.05	319.82	0.05
3	Pentane, 2-methyl-	C6H14	381.85	6.95	349.95	3.26	340.82	1.02
4	β-Pinene	C_10_H_16_	364.80	3.83	364.87	4.30	364.58	3.07
5	Sabinene	C_10_H_16_	380.90	4.74	380.90	4.69	380.78	5.71
6	Succinic anhydride	C_4_H_4_O_3_	384.46	1.29	386.12	1.37	385.98	0.92
7	3-Carene	C_10_H_16_	410.02	0.05	410.24	0.36	410.05	0.15
8	β-Myrcene	C_10_H_16_	428.93	1.53	429.20	2.90	429.03	2.25
9	Terpinene	C_10_H_16_	443.21	0.15	444.32	0.18	444.30	0.11
10	Limonene	C_10_H_16_	466.15	24.29	464.81	33.25	462.81	26.34
11	D-Limonene	C_10_H_16_	462.75	29.89	468.73	26.45	464.89	37.20
12	β-Phellandrene	C_10_H_16_	472.71	0.28	474.30	0.21	473.29	0.08
13	γ-Terpinene	C_10_H_16_	508.93	13.61	509.01	9.29	508.99	10.11
14	O-Cymene	C_10_H_14_	nd	nd	533.46	0.10	533.64	0.17
15	Octanal	C_8_H_16_O	550.88	0.31	550.95	0.23	550.93	0.39
16	Nonanal	C_9_H_18_O	642.98	0.32	642.85	0.31	642.75	0.44
17	P-Mentha-1,5,8-triene	C_10_H_14_	671.25	0.01	671.25	0.01	671.22	0.10
18	Acetic acid, octyl ester	C_10_H_20_O_2_	707.24	0.03	691.65	0.05	707.32	0.08
19	Trans-Limonene Oxide	C_10_H_16_O	696.50	0.08	696.48	0.15	696.49	0.09
20	Citronellal	C_10_H_18_O	712.53	0.06	712.48	0.09	712.50	0.11
21	Copaene	C_15_H_24_	723.66	0.06	723.69	0.18	723.86	0.07
22	Decanal	C_10_H_20_O	727.75	0.04	728.07	1.35	727.82	0.98
23	Linalool	C_10_H_18_O	762.91	1.86	762.72	2.08	762.82	2.88
24	Trans-α-Bergamotene	C_15_H_24_	781.20	0.01	nd	nd	794.49	0.03
25	β-Elemene	C_15_H_24_	797.98	0.03	798.17	0.08	799.05	0.04
26	β-Sophorene	C_15_H_24_	800.90	0.24	800.95	0.25	802.32	0.14
27	Caryophyllene	C_15_H_24_	805.35	0.27	805.25	0.30	806.69	0.16
28	Undecanal	C_11_H_22_O	807.30	0.27	807.24	0.10	nd	nd
29	Cis-β-Farnesene	C_15_H_24_	814.28	0.01	849.37	0.09	nd	nd
30	Neral	C_10_H_16_O	864.58	0.36	864.48	0.31	864.46	0.15
31	α-Terpineol	C_10_H_18_O	876.67	1.27	876.72	1.46	876.67	1.57
32	Dodecanal	C_12_H_24_O	882.16	0.13	882.32	0.25	882.31	0.19
33	Valencene	C_15_H_24_	nd	nd	891.56	0.39	891.67	0.32
34	β-Bisabolene	C_15_H_24_	894.23	0.27	894.19	0.20	nd	nd
35	Citral	C_10_H_16_O	899.27	0.85	899.22	0.65	899.26	0.36
36	Carvone	C_10_H_14_O	903.32	0.16	903.34	0.30	903.34	1.03
37	Nerol	C_12_H_20_O_2_	911.71	0.49	911.70	0.09	911.69	0.03
38	Perillaldehyde	C_10_H_14_O	938.88	0.07	938.90	0.11	938.86	0.15
39	(E,E)-2,4-Decadienal	C_10_H_16_O	952.82	0.01	952.86	0.02	952.82	0.02
40	D-piperonone	C_10_H_14_O	974.30	0.01	974.31	0.04	974.28	0.07
41	Perilla acetate	C_12_H_18_O_2_	981.35	0.05	981.30	0.04	981.52	0.05
42	1-Decanol	C_10_H_22_O	995.71	0.15	995.17	0.46	994.94	0.27
43	Geranyl formate	C_12_H_18_O_2_	1037.23	0.38	1036.80	0.25	nd	nd
44	Caryophyllene oxide	C_15_H_24_O	1065.51	0.01	1065.47	0.02	1065.49	0.01
45	Phenol	C_6_H_6_O	1074.46	0.35	1074.59	0.03	1074.59	0.03
46	Methyleugenol	C_11_H_14_O_2_	1076.64	0.08	1076.48	0.01	1076.64	0.01
47	Octanoic acid	C_8_H_16_O_2_	1102.17	0.04	1101.15	0.04	1065.49	0.01
48	Elemol	C_15_H_26_O	1121.80	0.01	1121.61	0.02	1121.61	0.03
49	Perilla alcohol	C_10_H_16_O	1129.28	0.06	1128.62	0.08	1137.89	0.06
50	Nonanoic acid	C_9_H_18_O_2_	1163.47	0.02	1162.61	0.02	1162.92	0.03
51	Thymol	C_10_H_14_O	nd	nd	1176.26	0.01	1193.46	0.01
52	Sinensal	C_15_H_22_O	1202.08	0.01	1202.14	0.02	1202.12	0.02
53	N-Decanoic acid	C_10_H_20_O_2_	1221.70	0.01	1220.84	0.02	1221.18	0.03
	Sum (in % total)			98.13		99.12		99.31

Note: HD conditions: temperature 100 °C, duration 3 h, solid–liquid ratio 1:20; OH conditions: electric field 25 V/cm, temperature 100 °C, duration 60 min, solid–liquid ratio 1:20; EH conditions: oscillating magnetic field 1.39 T, temperature 100 °C, duration 60 min, solid–liquid ratio 1:20. nd means not detected.

## Data Availability

The original contributions presented in the study are included in the article, further inquiries can be directed to the corresponding author.
